# Genetic structure of immunologically associated candidate genes suggests arctic rabies variants exert differential selection in arctic fox populations

**DOI:** 10.1371/journal.pone.0258975

**Published:** 2021-10-29

**Authors:** Tristan M. Baecklund, Michael E. Donaldson, Karsten Hueffer, Christopher J. Kyle

**Affiliations:** 1 Environmental and Life Sciences Graduate Program, Trent University, Peterborough, ON, Canada; 2 Department of Veterinary Medicine, University of Alaska Fairbanks, Fairbanks, AK, United States of America; 3 Forensic Science Department, Trent University, Peterborough, ON, Canada; 4 Natural Resources DNA Profiling & Forensic Centre, Trent University, Peterborough, ON, Canada; UCSI University, MALAYSIA

## Abstract

Patterns of local adaptation can emerge in response to the selective pressures diseases exert on host populations as reflected in increased frequencies of respective, advantageous genotypes. Elucidating patterns of local adaptation enhance our understanding of mechanisms of disease spread and the capacity for species to adapt in context of rapidly changing environments such as the Arctic. Arctic rabies is a lethal disease that largely persists in northern climates and overlaps with the distribution of its natural host, arctic fox. Arctic fox populations display little neutral genetic structure across their North American range, whereas phylogenetically unique arctic rabies variants are restricted in their geographic distributions. It remains unknown if arctic rabies variants impose differential selection upon host populations, nor what role different rabies variants play in the maintenance and spread of this disease. Using a targeted, genotyping-by-sequencing assay, we assessed correlations of arctic fox immunogenetic variation with arctic rabies variants to gain further insight into the epidemiology of this disease. Corroborating past research, we found no neutral genetic structure between sampled regions, but did find moderate immunogenetic structuring between foxes predominated by different arctic rabies variants. F_ST_ outliers associated with host immunogenetic structure included SNPs within interleukin and Toll-like receptor coding regions (IL12B, IL5, TLR3 and NFKB1); genes known to mediate host responses to rabies. While these data do not necessarily reflect causation, nor a direct link to arctic rabies, the contrasting genetic structure of immunologically associated candidate genes with neutral loci is suggestive of differential selection and patterns of local adaptation in this system. These data are somewhat unexpected given the long-lived nature and dispersal capacities of arctic fox; traits expected to undermine local adaptation. Overall, these data contribute to our understanding of the co-evolutionary relationships between arctic rabies and their primary host and provide data relevant to the management of this disease.

## Introduction

Hosts and pathogens are in a continual co-evolutionary arms race, where patterns of local adaptation can emerge in response to the selective pressures diseases exert on host populations, and thus influence disease spread and maintenance [1–3]. Elucidating where adaptations have occurred throughout the genomes of host populations can provide better understanding of disease mechanisms in general, including the impacts of pathogens in shaping host population diversity, and how perturbations to these systems may influence disease distributions and outcomes in host populations.

Divergent selection can lead to locally adapted populations across heterogeneous landscapes where selective pressures differ [1–3]. When divergent selection occurs without interference from other forces, local populations evolve traits best suited to local pressures providing increased fitness within a specific environment regardless of the consequences of the trait in different environments [1–3]. In natural populations, the process of local adaptation is largely influenced by three factors: gene flow, effective population size/genetic drift, and force of the selective pressure [1]. In these natural systems, homogenization of variation through gene flow and stochastic loss of variants via genetic drift can undermine increases in adaptive trait frequencies that are suggestive of local adaptation [1–3]. Thus, when trying to elucidate patterns of local adaptation in natural populations is it necessary to evaluate the effects of gene flow and genetic drift in context of the distribution and frequencies of traits under natural selection.

Patterns of local adaptation are often assessed through common garden experiments, where populations are exposed to a series of different environmental variables, and changes in fitness are observed over time [1]. In natural populations common garden experiments are not always feasible, and they are further complicated by plastic responses of populations, where single genotypes can elicit multiple phenotypes that mask measurable changes in fitness [4, 5]. Genetic assessments of the interplay between selection and demographic forces, such as by contrasting patterns of neutral genetic structure of populations relative to the genetic structure of loci under selection, can provide an alternate means to detect genetic signatures indicative of local adaptation in lieu of common garden experiments. Examples of this approach include assessments of the variation of salmonid immune responses within environments with different aquatic thermal regimes [6] and variation in genes associated with vision and hearing in wolf populations in context of specific environmental variables [7].

While species are exposed to a myriad of selective pressures, infectious diseases can exert strong selective pressures over short periods of time, where the genetic composition of a population can change considerably within a few generations from these selective sweeps [8, 9]. The emergence of white-nose syndrome in bats [10, 11] and the development of facial tumors in Tasmanian devil populations [12], where both diseases led to drastic population declines, are exemplar of the rapid effects infectious diseases can have on natural populations. In some patho-systems, such as chronic wasting disease (CWD) in mule deer, population differences in the frequency of genotypes responsible for susceptibility to CWD have been observed, providing strong evidence for local adaption to this disease over time [13]. Patterns of host-pathogen interactions can also occur over longer time scales and lead to coevolutionary interactions when diseases are endemic and multiple variants of disease circulate in the environment. In these systems, host populations adapt to persist against pathogens and pathogens evolve to circumvent host immune defenses [2, 3]. Thus, a population’s response to disease can be dependent upon both genetic variants circulating within host populations, but also the genetic variants of the pathogen(s) host populations are exposed to [14].

Explorations of population responses to disease have typically undertaken genetic assessments of the major histocompatibility complex (MHC) due to its association with antigen binding and its highly polymorphic nature [15–18]. Specifically, MHC DRB exon-2 has been used extensively as an indicator of the genetic variation of MHC [19, 20], yet studies have indicated that variation in other genomic areas associated with an immune response also play important roles in defenses against infectious disease [21–24]. Genotyping-by-sequencing (GBS) assays enable assessments of genetic variation from a larger number of loci simultaneously, thus providing an opportunity to explore the variation of genes associated with an immune response more holistically [7, 25, 26]. For example, GBS has been used to explore 138 genes associated with the immune response in little brown bats in context of white-nose syndrome exposure [27], and Elbers et al. [28] found immunologically relevant variants associated with macromolecule and protein modifications in gopher tortoises that influenced upper respiratory tract disease severity. In the absence of feasible/pragmatic common garden experiments, GBS techniques provide a means to study the genetic impacts disease can have on host populations.

**Fig 1:**
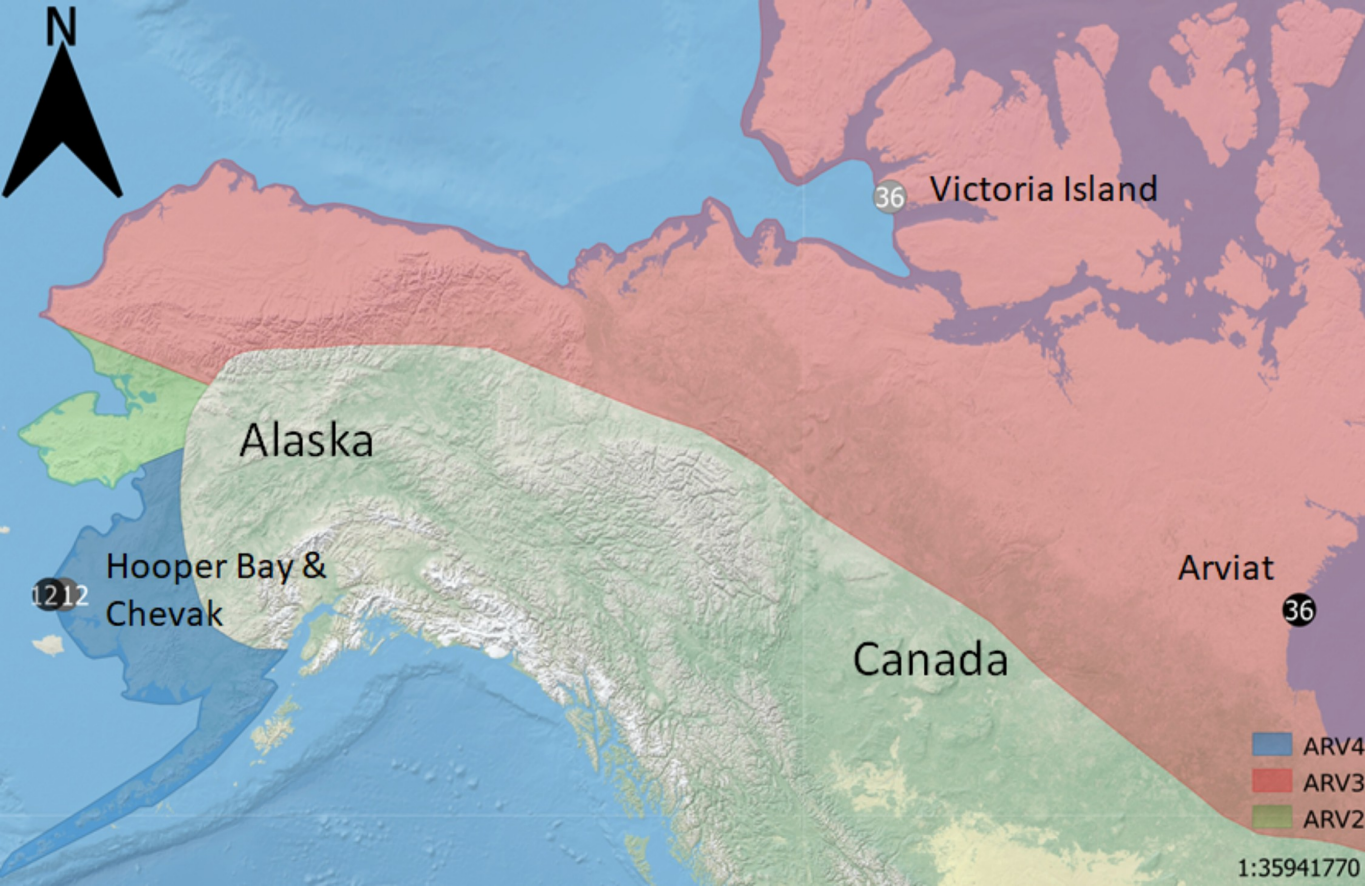
Approximate arctic rabies variant distributions in North America and schematic of the 96 arctic fox samples used in the genotype-by-sequencing assay. Circles indicate sample locations; numbers within circles indicate sample size. Samples were obtained from Arviat, NU (n = 36); Victoria Island, NWT (n = 36); and two regions of Southwest Alaska, (Chevak (n = 12) and Hooper Bay (n = 12). Approximate arctic rabies viral variant distributions (ARV 2,3,4) are depicted by colored regions (see in-figure legend). The schematic of viral variant distributions was adapted from Goldsmith et al., 2016 [31] for illustrative purposes only. Map created using Natural Earth (naturalearthdata.com).

Arctic rabies (AR) is a lethal lyssavirus that circulates in northern climates through its natural host, the arctic fox (*Vulpes lagopus*), where epizootic cycles of the disease occur every 3–6 years [29, 30]. Arctic rabies is comprised of four phylogenetically distinct subvariants, where all four variants circulate in unique geographically maintained distributions in North America (Fig 1) [30–33]. Arctic rabies variant 2 (ARV2) is restricted to the Seward Peninsula of Alaska, AR variant 4 (ARV4) is restricted to Southwestern Alaska, and AR variant 3 (ARV3) circulates along northern coasts across North America and Eurasia [31]. However, AR variant 1 (ARV1) circulates only in Southern Ontario and is maintained in the absence of arctic fox, presumably by red fox [34]. Despite these phylogenetic and distribution differences, it remains unknown whether geographically restricted AR variants have differences in pathogenicity that may impose divergent selection between arctic fox populations, and potentially reveal signatures of locally adapted host populations.

In Alaska, three variants of AR circulate predominantly within red and arctic fox populations [31]. Previous red fox studies found that patterns of neutral genetic structure [31] and genetic variants associated with an immune response [35] demonstrated correlations of red fox genetic structure with the presence/absence of AR. However, no signatures indicative of differential selection were observed between AR variants in the red fox system. In contrast, the observed neutral genetic structure of arctic fox was noted to closely parallel the distribution of AR variants [31]. These data were corroborated by the fact that where AR variant 3 persists throughout northern coasts, arctic fox exist as a largely panmictic population as a matter of high gene flow facilitated by sea ice connectivity [36–38]. While inferences of disease spread/maintenance through observations of patterns of host gene flow based on neutral genetic markers are feasible, neutral markers alone do not provide insight into coevolutionary patterns that may exist between arctic fox and AR. Therefore, assessments of genetic variation associated with responses to selective pressures exerted by AR, such as genes related to an immune response, have the potential to further our understanding AR spread and maintenance in arctic fox populations.

Herein, we build upon previous research [31, 35] using an immunogenetic assay targeting 116 regions of the arctic fox genome associated with an immune response. We aimed to: 1) determine if genetic variants associated with an immune response give rise to patterns of genetic structure in arctic fox, and 2) determine if patterns of differential selection exist in artic fox relative to AR variant distributions that may be indicative of local adaption to this disease; data that also provides insight into the maintenance and spread of AR. The Arctic continues to experience rapid warming, thus understanding host population responses to different disease variants, and the potential for local adaptation in hosts, becomes increasingly important as climatic changes are expected to cause range shifts in both pathogens and their hosts [29, 30, 39–43]. Overall, this research aims to enhance our understanding of AR dynamics in arctic fox where unique distributions of AR variants are maintained in North America.

## Methods

### Sampling, DNA extractions and quantification

Arctic fox muscle tissue samples were obtained from various independent trappers and organizations as part of the University of Alaska Museum of the North tissue collections or as part of previous research [36; S1 Table]. No direct handling/sampling of animals took place for this study. Samples were stored at -80°C until processed. Samples were digested in 200 μL 1X lysis buffer (4 M Urea, 0.2 M NaCl, 0.5% n-lauroyl sarcosine, 10 mM ethylenediaminetetraacetic acid (EDTA), 0.1 M Tris HCl pH 8.0) with the addition of 20 μL proteinase K and incubated at 56°C for two hours. During digestion, samples were vortexed and briefly spun down every 30 minutes. DNA was extracted from the resulting lysate utilizing the DNeasy Blood and Tissue Kit (Qiagen) following manufacturer protocols with the exception that DNA was eluted in a total volume of 60 μL, using two 30 μL aliquots of TE buffer (10 mM Tris, 0.1 mM EDTA). Isolated DNA was quantified using the Quant-iT PicoGreen dsDNA Assay Kit (ThermoFisher Scientific) and quality assessed by ethidium stained 0.8% agarose gel electrophoresis (90 V for 45 minutes) where DNA fragment size was assessed in context of HighRanger 1 kbp DNA ladder (Norgen Biotek). A subset of 96 high molecular weight DNA samples suitable for sequencing were selected from three regions across the arctic fox’s distribution in North America (Fig 1; S1 Table). Samples from Hooper Bay and Chevak are referred to as a single region ‘Southwestern Alaska’ based on their geographic proximity to one another but were left ungrouped for neutral genetic analyses (~ 30 kilometers).

### Library preparation, sequence capture and high-throughput sequencing

DNA libraries were prepared using Kapa HyperPlus Kit (Roche) following the SeqCap-EZ HyperCap UGuide V1.0 (Roche) protocol. Seven cycles were implemented as part of the pre-LM PCR as recommended by the manufacturer with the following modifications to the workflow: i) PCR-grade water was used for dilutions and elution, ii) samples were treated with 5 μL of conditioning solution during fragmentation, iii) TruSeq HT Dual-Index Adapters (Integrated DNA Technologies) were used in place of SeqCap Adapter Kits A and B (Roche), and iv) Illumina P5 and P7 primers (Integrated DNA Technologies) were substituted in place of the Pre LM-PCR Oligos 1 & 2 (Roche). At the end of the Pre-capture LM-PCR step, DNA library quality was assessed using ethidium bromide-stained gel electrophoresis as per above.

A 1 μg DNA multiplex was created from equal-molar amounts of each of the 96 libraries. Target enrichment was performed as previously described [35], using the designed SeqCap EZ Developer Library probe. Modifications to the enrichment protocol implemented in this study included: i) replacement of the NimbleGen Multiplex Hybridization Enhancing Oligo Pool (Roche) with 2 μL xGen Universal Blockers–TS Mix (Integrated DNA Technologies), ii) NimbleGen SeqCap EZ Developer Reagent (Roche) was used in place of the NimbleGen COT Human DNA (Roche) during hybridization sample preparation, and iii) hybridization was carried out at 47°C for 20 hours. A final product assessment was conducted with a bioanalyzer on the target-enriched multiplex before sequencing on an Illumina MiSeq V3 run using 2x300 bp reads (Advanced Analysis Centre Genomics Facility, University of Guelph).

### Sequence alignment and variant annotation

Utilizing the bwa-mem command in Burrows-Wheeler Aligner v0.7.12 [44], paired-end reads for each of the 96 samples were aligned to the canine reference genome (CanFam3.1; Fig 1; S1 Table). After sequence metrics were obtained using SAMTOOLS v1.5 [45], the Genome Analysis Toolkit (GATK, V4.0.0.0) best practices pipeline and standard hard filtering parameters were used to perform duplicate sequence removal, SNP/INDEL variant annotation, genotyping, and variant recalibration [46–48]. The SelectVariants function was then used to compile a VCF file containing only bi-allelic SNPs.

The SeqCap EZ Developer Library probe was originally designed from a draft version of the red fox genome [33], thus positions of the probe-baited targets needed to be determined and converted into positions in the canine reference genome (CanFam3.1; Accession: PRJNA12384) using BLASTn (S2 Table). These targeted regions were compiled into a list of on-target intervals. On-target intervals were used to further categorize SNPs as being within coding regions (including 1,500 bp upstream from the start codon) or within intergenic (off-target; outside targeted coding regions and promoters) regions. We attempted to mitigate biases to identify loci under selection using F_ST_ outlier tests as recommended in the literature [49] by accounting for: linkage disequilibrium within datasets, method variation by implementing several F_ST_ outlier tests, and filtering variants for minimum allele frequency (MAF).

### SNP filtering and analyses

Both sub datasets of SNPs from within coding regions, and those from intergenic regions were filtered using VCFtools v0.1.13 to retain only biallelic variants with a MAF threshold of 2.5%, a maximum missing genotype threshold (per site) of 20% and excluding variants on the X-chromosome. Additionally, both filtered sub-datasets were pruned for linkage disequilibrium as implemented by the SNPRelate package in R v.3.5 [50, 51] and further pruned for physical linkage to only retain SNPs ≥ 100kbp from one another using bcftools v1.9 [52] (S1 File for further details). SNPs from within intergenic regions underwent further filtering using the Ensembl Variant Effect Predictor [53] to retain only those SNPs ≥ 40 kbp from an annotated coding region. Disequilibrium and physical linkage pruning occurred after F_ST_ outlier tests for the sub-dataset composed of SNPs from within coding regions. Mantel tests (as implemented in R) [50] were performed on both the intergenic SNP-dataset and the SNP-dataset from coding regions to identify patterns of isolation-by-distance.

#### Analyses of SNPs in intergenic regions

Variants passing filtering parameters set for the intergenic regions were assumed to not be under selective influence and were therefore used to estimate patterns of neutral population genetic structure. The filtered sub-dataset was analyzed using principal component analysis (PCA) and discriminant analyses of principle components (DAPC) as implemented in RStudio using the adegenet (v2.1.1) [54] and ape (v5.1) [55] packages. Principle components with eigenvalues ≥ 0.1 were retained for the PCA, and cross validation was used to determine the number of retained components based on the root mean squared error (lowest MSE) for the DAPC. Discriminant analyses implemented successive K-means to determine the optimal number of identified clusters of the data.

Implementing STRAUTO (v.1.0) [56], STRUCTURE analyses were performed with a burn-in length of 50,000 followed by 200,000 iterations for K = 1 through K = 6, with 20 iterations of each K. Using structure harvester web (v0.6.94) [57], the ΔK statistic was calculated to determine the number of distinct genetic clusters inferred from the data. CLUMPP v1.1.2 [58], and the LargeKGreedy algorithm (10,000 repeats) were used to assign individuals to genetic clusters, followed by the implementation of DISTRUCT v1.1 [59] to combine and visualize results.

POWSIM v.4.1 [60] was used to estimate the effective power of the presumed neutral SNP sub-dataset to detect genetic structure. Simulations were run with Ne = 500 and 5,000, t = 0, 10, 100, 500, and 1,000. Each set of conditions was performed 1,000 times to differentiate between the three sampled regions. A Fisher’s exact test was implemented within the program using a Monte Carlo Markov chain approach with default parameters of 1,000 burn-ins, 100 batches, and 1,000 iterations.

#### Analyses of SNPs in coding regions

Variants within coding regions that passed initial MAF and missing data filtering parameters were assessed with PCAdapt [61], OutFLANK [62], Arlequin [63], and Bayescan [64] to identify F_ST_ outliers within the sub-dataset. Each of these tests identified outliers using an adjusted p-value threshold of ≤ 0.05; more detailed parameters for each method can be found as a supplement (S1 File). Inconsistencies in identified outliers can occur between different methods due to differences in underlying assumptions and caveats used by each method [62]. For the purposes of this study, we retained any outlier identified by at least one of the methods in the final sub-set of SNPs from within coding regions. The final sub-set of outliers was then pruned for linkage disequilibrium and physical linkage prior to PCA, DAPC, and structure analyses, as described above. Further, to provide an adequate control for these data, we attempted to repeat F_ST_ outlier detection on the off-target dataset utilizing the same methods and parameters as the on-target data. F_ST_ estimates were generated using VCFtools (based on Weir & Cockerham, 1984), and 97.5% F_ST_ confidence intervals were determined in R.

The program SnpEff was used to annotate synonymous and non-synonymous polymorphisms within the dataset of SNPs from coding regions (using the CanFam3.1.99 database) [65]. Using these annotations, we calculated the relative ratio of non-synonymous substitutions per non-synonymous site to the number of synonymous substitutions per synonymous site (pN/pS) as highlighted by Nei and Gojobori; $pN=\frac{Nd}{N}$ and $pS=\frac{Sd}{S}$ [66]. Where Nd/Sd is the number of nonsynonymous or synonymous polymorphisms and N or S is the total number of nonsynonymous or synonymous sites [66, 67]. Following previous research, we determined the potential number of nonsynonymous/synonymous sites using DnaSP v6 and the coding sequence for each gene as input [67, 68] where positive selection can be inferred from pN/pS ratios > 1, and purifying selection from ratios < 1 [69].

## Results

### Raw sequence data

We obtained an average of ~ 563,000 raw reads per library, 99.5% of which mapped to the canine reference genome. After processing the raw data through the GATK SNP calling pipeline, an average of ~ 109,000 (19.35%) reads were filtered from each sample library leaving ~ 452,000 reads per library. An average of 56% reads per library mapped to probe targeted regions with an average of 54 X coverage across all 96 libraries (S3 Table).

### SNPs in intergenic (off-target) regions

**Fig 2:**
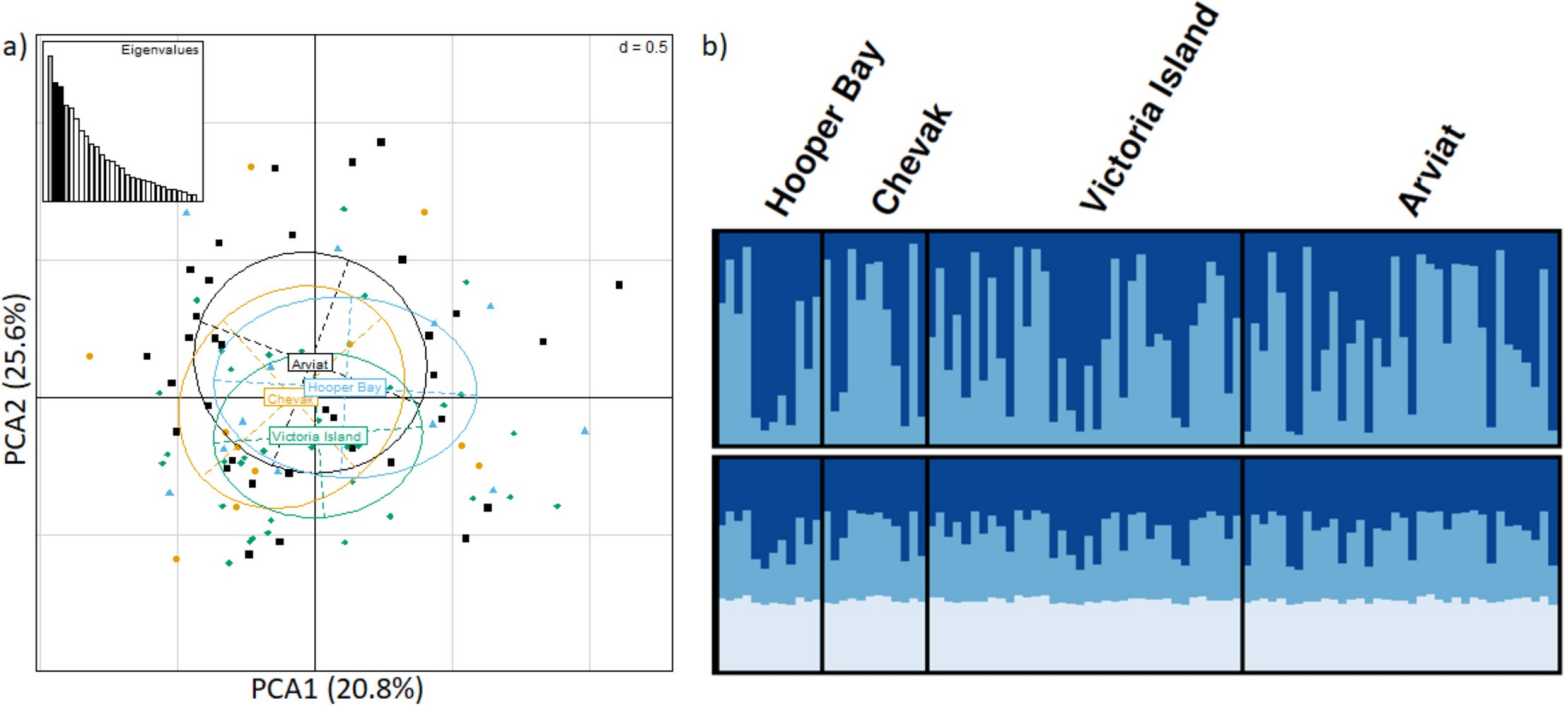
Neutral genetic homogeneity of arctic fox across North America. Analyses of the 29 presumed neutral SNPs after filtering with Variant Effect Predictor, MAF = 2.5%, and pruning for Linkage disequilibrium **A)** principle component analysis **B)** STRUCTURE analysis of K = 2 and 3 (top and bottom respectively), where each bar across horizontal axis indicates an individual, vertical axis depicts cluster assignment, and different colors depict each genetic cluster; Arviat = black square; Chevak = yellow circle; Hooper Bay = blue triangle; Victoria Island = green diamond.

A dataset of 5,490,704 intergenic SNPs was filtered using Variant Effect Predictor, which was then pruned to minimize linkage disequilibrium. This yielded a dataset of 29 intergenic SNPs presumed to be not under selective pressure with an average depth of coverage of 15 X across all SNPs (average depth of coverage of 5 X when excluding 4 SNPs with coverage exceeding 15X), and a MAF of 2.5% (S4 Table). These data were visualized using PCA, DAPC and STRUCTURE. PCA did not identify genetic structuring as all clusters had extensive overlap with each other (S9 Fig). The DAPC identified K = 6 as the most likely number of clusters; however, STRUCTURE analyses of K = 2–6 showed high levels of admixture across all analyses and indicated an optimal K = 2 clusters (Fig 2). Power analyses of these 29 SNPs indicated a power of ~86% at an expected F_ST_ of 0.01 and a power of 100% at an expected F_ST_ of 0.05 or above, indicating a high likelihood that if population differentiation was > 1%, our dataset had the power to detect that difference. Isolation-by-distance was not observed within intergenic SNPs based on Mantel tests. We observed a total of 4 outlier SNPs (13 prior to linkage pruning) within the off-target dataset, all of which were identified by PCAdapt, and visualization of these data demonstrate no genetic clustering (S8 Fig). Overall, these analyses indicated no apparent genetic structure across the sample design for the off-target, and presumed neutral, SNP dataset.

### SNPs in coding (on-target) regions

**Fig 3:**
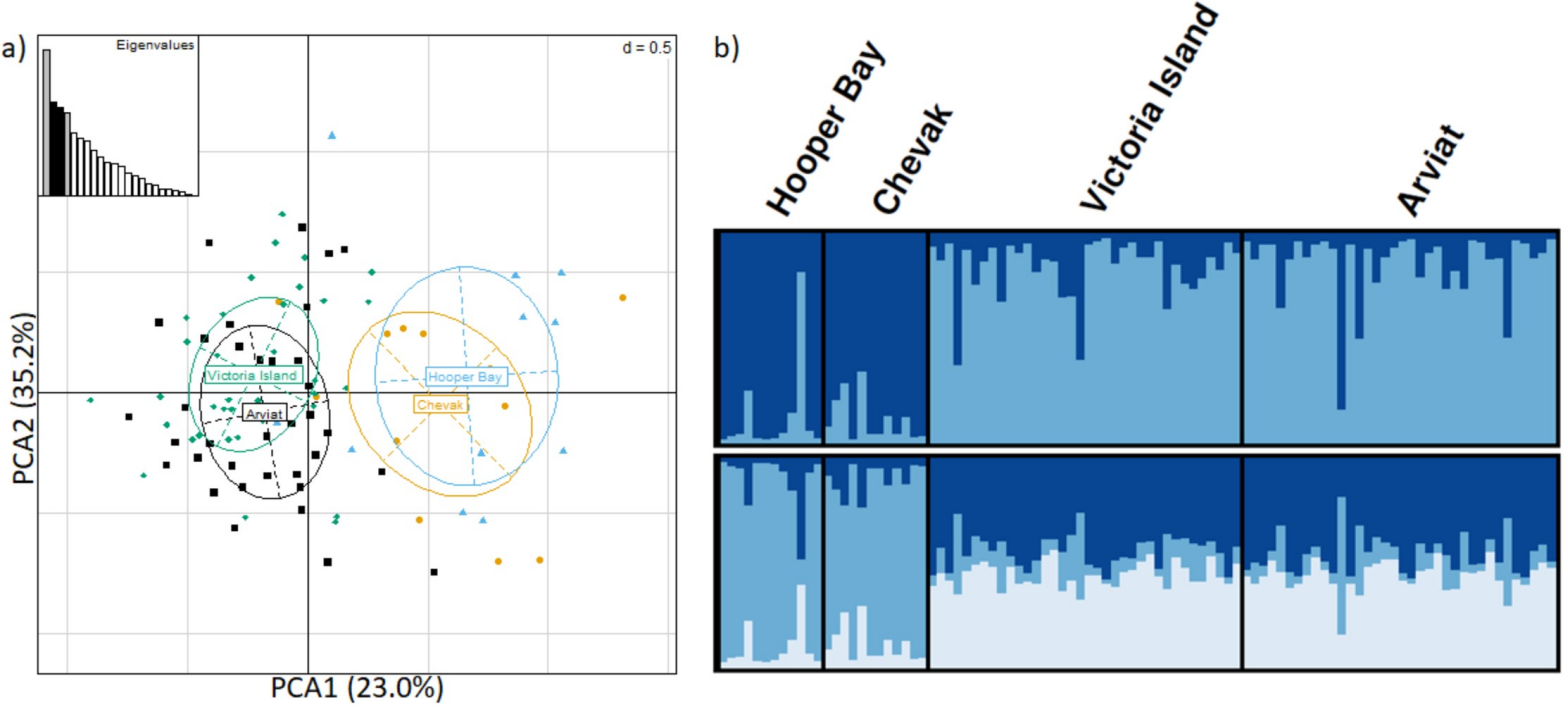
Arctic fox immunogenetic structure differentiates sampled regions in Southwestern Alaska and Northern Canada. Analyses of 22 protein-coding F_ST_ outlier SNPs after filtering for MAF = 2.5% and pruning for Linkage disequilibrium **A)** principle component analysis and **B)** STRUTURE analysis of K = 2 and 3 (top and bottom respectively), where each bar across horizontal axis indicates an individual, vertical axis depicts cluster assignment, and different colors depict each genetic clusters; Arviat = black square; Chevak = yellow circle; Hooper Bay = blue triangle; Victoria Island = green diamond (note, Chevak and Hooper Bay were pooled for this analysis).

We found 9,467 SNPs located within target intervals before filtering. After applying search criteria for 2.5% MAF, a maximum of 20% missing data, and discarding SNPs on the X-chromosome, 2,277 SNPs remained. F_ST_ outlier analyses on these 2,277 SNPs produced a sub-dataset containing 107 SNPs (S5 Table). After accounting for linkage disequilibrium, a final sub-dataset containing 22 F_ST_ outlier SNPs remained, several of which were associated with interleukins, Toll-like receptors, and the MHC (S5 Table). Based on Variant Effect Predictor results, four SNPs retained in the final sub-dataset associated with DLA-DQA, NOD1, RAG1 and TLR5 genes, were likely to convey a change in chemical characteristic of the encoded amino acid during translation (e.g., acidic to basic amino acid). A further 9 SNPs, that conveyed such missense changes, were removed from the final sub-dataset when filtering for linkage disequilibrium (S5 Table). DAPC and STRUCTURE analyses of the remaining 22 F_ST_ outlier SNPs were assessed identifying K = 2 clusters. We found arctic fox sampled from Alaska appeared genetically distinct from foxes from Northern Canada (Fig 3) with F_ST_ estimates of 0.127. F_ST_ confidence intervals indicated that F_ST_ between Southwestern Alaska and the two regions in Canada were significantly different from zero; however, F_ST_ between the two Canadian arctic fox populations was not significantly different from zero (S6 Table). It is important to note however, that we could not exclude isolation-by-distance as a potential factor contributing to these patterns (could not be calculated being only 2 geographic points–Canadian vs Alaskan samples).

Estimates of pN/pS were determined for 90 of the initial 116 targeted genes (S7 Table) although for 17 of these genes, pN/pS ratios could not be calculated due to a lack of polymorphic substitutions (either nonsynonymous or synonymous) resulting in pN values equal to zero or division by zero errors (occurring when pS = 0). The pN/pS ratios for most genes were indicative of purifying selection; however, 10 genes (C2, DLA-12, DLA-79, DLA-DQA, DLA-DBQBC1, IL1B, IL23A, MYD88, STAT3, and STAT6) appeared to be under positive selection (pN/pS ratio ≥ 1) in all three arctic fox populations sampled (S7 Table). One gene appeared to be under positive selection only in Southwestern Alaska (CD8A), whereas both CCL2 and CCR8 were under positive selection within foxes sampled from Canada (Arviat and Victoria Island; S7 Table). Based on an assessment of Chi^2^ p-values (S7 Table) only the STAT3 gene had a p value of p < 0.05 (0.000137), indicating a significantly different pN/pS ratio from expected values.

## Discussion

Herein, we contribute to the understanding of host/pathogen evolutionary systems by examining the genetic structure of immunologically associated molecular markers of arctic fox in context of arctic rabies variants. Specifically, we used a genotyping-by-sequencing (GBS) assay to explore immunogenetic regions of the arctic fox genome that also yielded off-target sequence data from intergenic regions presumed to be neutral. We take these presumed neutral data, combined with immunogenetically relevant data, to suggest patterns of local adaptation exist in this system as expected if AR variants displayed differential selection upon arctic fox populations across their range. While these correlations do not necessarily reflect causation, AR is known to present strong selective forces on arctic fox, combined with the observed structure at several identified F_ST_ outliers associated with genes known to mediate response to rabies [70–73] in a presumably panmictic population (37–39; and data presented herein), is notable. Further, pN/pS ratios identified genes under positive selection in arctic fox across North America, but also between sampled regions where different AR variants circulate. As only one gene provided a significantly different pN/pS ratio from expected values (S7 Table), these data should be interpreted with caution given previous research highlighting that these analyses should not be interpreted as sufficient evidence of selection on their own [74, 75] and are subject to several biasing factors, such as few polymorphisms identified within genes [69, 76]. We take these data to imply that patterns of local adaptation exist in the arctic fox/rabies system reflecting the strong selective pressure AR likely exerts on arctic fox populations despite the impressive dispersal abilities and panmictic neutral genetic structure of arctic fox [31, 36–38]. While these data provide insight into how unique distributions of AR variants are maintained, potential differences in pathogenicity between AR variants have not been established and further research is required to ascertain more definitive insight into the interrelationship of AR variants and their main host. Specifically, research encompassing more arctic foxes from across their range that include all AR variant distributions accompanied by further evidence of underlying pathogenic differences between variants would benefit our understanding of AR maintenance and spread.

### Analyses of intergenic SNPs (off-target)

The generation of secondary (off-target) sequences using targeted GBS approaches is a common feature observed in other studies [77]. These untargeted products are often consistently sequenced allowing these data to be used in downstream analyses if they are of sufficient coverage and quality [77–79]. Studies have noted that simulation-based assessments should be combined with off-target datasets to determine the power of these data to discern differences between populations and therefore form meaningful conclusions [80, 81]. Although the observed neutral genetic structure presented here is devoid of the subtle structure previously found among arctic fox populations in Alaska (F_ST_ = 0.02 with microsatellites) [31], it remains consistent with coarse geographic scale studies of the species [36–38]. This is unsurprising to a limited extent given foxes from only the northern coast of Alaska were used as representatives for the whole state in these coarse-scale assessments as previously noted by Goldsmith et al. [31]. It is worth noting that the inability to detect similar patterns of neutral population genetic structure in this study were likely due to the limited number of variants passing the filtering parameters, combined with differences in the power of discrimination between microsatellites and biallelic SNPs. Furthermore, when we attempt to identify F_ST_ outliers within the off-target dataset, there were insufficient numbers of outliers (4 after linkage pruning) to draw any meaningful result. The lack of identified outliers likely results from prominent gene flow documented among arctic fox, and as such, in conjunction with data presented in these previous studies [31, 36–38], we take the presumed neutral data presented herein to provide further evidence of the extensive gene flow among North American arctic fox populations.

While we acknowledge that filtering parameters employed herein were rigorous and likely removed informative SNPs form the final subsets used in analyses, determining neutral genetic structure of arctic fox across North America was not a direct objective of this study having been investigated thoroughly elsewhere [31, 36–38]. Additionally, recent research suggests recombination distances vary across chromosomes in red foxes (ranging from 0.07 cM– 5 cM between pericentromeric regions and chromosome ends) [82], meaning that the filtering step to ensure these SNPs were not in proximity (≥ 40 kbp) of any annotated coding regions provides only a coarse approximation of neutrality at these sites. With these potential limitations acknowledged, we used off-target data to provide a contextual baseline for the on-target data.

### Analysis of SNPs in protein-coding regions

Multiple F_ST_ outlier identification programs were implemented to identify SNPs within genomic regions of interest in order to mitigate discordance between methods [62]. Of the 107 identified F_ST_ outlier SNPs within protein-coding regions, only 32 were identified by multiple programs, and only two were identified by all four methods used. The number of identified F_ST_ outliers between programs ranged from 9–61, where Bayescan identified the least and PCAdapt identified the largest number of F_ST_ outliers. Most identified F_ST_ outlier SNPs resulted in synonymous changes at their respective positions in the genome and thus unlikely to affect function of synthesized proteins. In contrast, 13 identified F_ST_ outlier SNPs conveyed a missense mutation that also changed chemical characteristics of translated amino acids, increasing the likelihood that these mutations could affect subsequent protein function [83]. These 13 missense F_ST_ outlier SNPs were associated with gene sequences of DLA-DQA, DLA-DQBC1, NOD1, RAG1 and TLR5, although only SNPs associated with the latter four genes were retained in the final filtered, linkage pruned, sub-dataset of 22 F_ST_ outlier SNPs. Both DLA-DQA and DLA-DQBC1 are class II components of the dog leukocyte antigen, responsible for initiating the immune response through antigen presentation and recognition [84]. NOD1 recognizes gram-negative bacteria and initiates a pro-inflammatory response [85], and RAG1 is a factor initiating immunoglobulin V(D)J recombination [86]. Finally, TLR5 detects bacteria with flagellin and induces a pro-inflammatory response [87]. Of interest are the large number of F_ST_ outlier SNP associations to interleukin and Toll-like receptor gene families, especially in context of AR, as members of these gene families are implicated in mediating a response to rabies [70–73]. Further, three missense F_ST_ outlier SNPs were associated with TLR5 demonstrating that this gene may play a prominent role in mediating responses to AR variants, although specific mechanisms of this response require further investigation.

Analyses of signatures of selection through assessments of pN/pS ratios demonstrate several genes that may be related to the spatially distinct distributions of AR variants. The CD8A gene is uniquely under positive selection in Southwestern Alaska, where AR variant 4 circulates. However, genes CCL2 and CCR8 appear to be under positive selection in both sampled regions in Canada where different AR variant 3 circulates. These data suggest that these three genes may be under positive selection, indicative of differential selection, relative to the AR variants in those locations and may play a role in the mechanisms that maintain unique distributions of AR variants. Of the ten genes under positive selection in all three populations, a large portion are involved in the MHC, which is expected given this gene family’s involvement in antigen binding and overall health of organisms [15–18]. Furthermore, two genes under selection in all three populations are associated with interleukins, a gene family already implicated in mediating a response to rabies [70, 71]. It is important to note that estimates of pN/pS are subject to potential biases in systems where there is prominent gene flow and migration [67], as is the case of arctic fox across their range, and by genes where there are few polymorphisms identified [69, 76]. To mitigate these potential biases, some researchers implement a threshold of pN/pS ratios > 2 to be indicative of positive selection [67]. In this context, only the STAT3 gene would appear to be under positive selection across all three of our sampled populations (pN/pS ratio of ~3.4). This gene was also the only gene to have a statistically significant pN/pS ratio with a p value of p < 0.05 (0.000137). However, in combination with the potentially inflated ratio of STAT3 due to those biases mentioned above, there were no outlier SNPs detected within the STAT3 gene. As such, although this gene may be under directional selection, it is unlikely to be contributing to the patterns of genetic structure observed within the on-target SNP-dataset. This is contrasted by outlier SNPs with the potential to change protein function identified within the genes DLQ-DQA and DLQ-DQBC1, however, these genes lacked significance in their pN/pS ratios that were suggestive of positive selection (pN/pS > 1; S7 Table). Given these results and potential biases, we present these pN/pS ratios only as an initial estimate requiring further testing and that these pN/pS results should be interpreted with caution.

### Arctic rabies variant distributions and differential selection

Analyses of F_ST_ outlier SNPs demonstrate that genetic differentiation between arctic fox populations inhabiting an AR variant region were not significantly different from zero, however, genetic differentiation between arctic fox populations inhabiting regions where a different AR variant circulates were significantly different from zero. Given that arctic fox populations have been exposed to AR over a large time frame [88], there remains the potential for coevolutionary forces to have shaped patterns of differential selection between AR variants where analyses of pN/pS ratios and identified F_ST_ outlier SNPs within immunogenetically relevant genes tentatively support these observations.

Previous data [31, 35–38], combined with those presented herein, demonstrate that arctic fox populations are largely panmictic with prominent gene flow, yet still appear to be locally adapted to the different AR variants. It had been anticipated that elevated levels of gene flow among arctic foxes [31, 35–38] would homogenize AR variants across the landscape, precluding local adaptation. In addition, rabies incubation periods range from several days to several months [85], which would prevent movement of AR variants, and further undermine the spatially distinct AR distributions observed. Some authors have theorized that dispersal capabilities of rabid foxes are reduced [31], and thus maintain AR variants spatial distributions where populations then undergo differential selection and subsequent local adaptation. However, there are no data to suggest phylogenetically distinct AR variants elicit differential responses, making it unclear how the weak genetic structure of immunogenic variants observed elicit differential selection. It is of interest that despite differences in biogeography and geographic distances between arctic fox populations from Arviat and Victoria Island, where AR variant 3 circulates, these two populations of arctic fox show no genetic structure of either presumed neutral or functional markers associated with an immunogenetic response. Further, in arctic fox from Southwest Alaska, where AR variant 4 circulates, there appears to be no neutral genetic structure in context of the Arviat or Victoria Island populations. This pattern contrasts the genetic clustering of functional, immunogenetically relevant, markers distinguishing between those arctic fox populations from Canada relative to those in Alaska; suggestive of differential selection between arctic rabies variants 3 and 4. Overall, we take these data to suggest that AR variants may impose differential selective pressures on populations despite the impressive dispersal capabilities and gene flow within the primary host, arctic fox.

There remains potential that the observed patterns indicative of local adaptation between arctic fox and AR variants to have arisen from purifying selection or genetic drift, rather than directional selection as interpreted here [89], with drift being unlikely in a panmictic system with extensive gene flow. We attempted to account for purifying selection biasing our interpretation by providing pN/pS ratios for genes where calculations were possible. Given the pitfalls of targeted sequencing approaches, such as the difficulty of interpreting signatures of selection from genomic data and successfully targeting the most informative loci [89], we present only candidate genes suggestive of patterns of differential selection. Continued research, such as whole genome analyses or sequencing of identified candidate genes presented herein, will be required to provide more support for the identified patterns of local adaptation. Whole genome analyses would benefit these data, as it would facilitate the testing of whether the observed patterns of genetic structure documented herein are consistent among other regions of the genome or an artifact of sequencing a handful of genes from the genome. Further, future research that aims to investigate the interrelationship between arctic fox and AR should sample from more populations of arctic fox from each AR variant distribution as well as include multiple populations for each AR variant region where possible. Additionally, the inclusion of foxes that have survived exposure to each AR variant, as well as those succumbing to the disease would greatly enhance the study by allowing for more in-depth analyses between AR variant regions, and direct consequences/benefits of specific SNPs. Lastly, there remains the potential for unknown factors, beyond the scope of the objectives and methods implemented here to have identified them, that may better explain the observed patterns of genetic structure.

## Conclusions

Infectious diseases can pose strong selective pressures on populations. Important to our understanding of the spread and maintenance of such diseases are the underlying interactions between host and pathogen [1–3]. By studying genetic variation within host populations associated with the immune response, we increase our understanding of how infectious diseases shape populations over time through patterns of local adaptation. Data from this study are also relevant to wildlife disease management efforts for arctic rabies where range shifts are occurring for both arctic and red fox, key arctic rabies vectors, in a rapidly warming Arctic. It remains unknown how these range shifts will affect the distributions of arctic rabies variants in North America.

## Supporting information

S1 Fig Principal component analyses of progressively filtered off-target SNP datasets. Clustering of the 96 arctic fox samples based on on-target SNPs throughout filtering steps. a) PCA of all off-target SNPs after filtering for MAF, missing-data, and biallelic SNPs (n = 6432) and b) PCA of all identified off-target SNPs after analysis with Variant Effect Predictor and prior to linkage pruning (n = 283). Arviat = black square; Chevak = yellow circle; Hooper Bay = blue triangle; Victoria Island = green diamond.
(TIF)

S2 Fig DAPC of final off-target SNP sub-dataset identifies six clusters. Discriminant analysis of principal components on the final on-target SNP sub-dataset (n = 29). a) the clustering of the samples into six inferred clusters, and b) the clustering of the samples into the same six inferred clusters as in a), but individuals are identified based on the geographical region from which the sample originated; cluster 1 (black square) = Arviat; cluster 2 (yellow circle) = Chevak; cluster 3 (blue triangle)–Hooper Bay; cluster 4 (green diamond)–Victoria Island.
(TIF)

S3 Fig Principal component analyses of progressively filtered on-target SNP datasets. Clustering of the 96 arctic fox samples based on on-target SNPs throughout filtering steps. a) PCA of all on-target SNPs after filtering for MAF, missing-data, and biallelic SNPs (n = 2277) and b) PCA of all identified on-target F_ST_ outlier SNPs prior to linkage pruning (n = 107). Arviat = black square; Chevak = yellow circle; Hooper Bay = blue triangle; Victoria Island = green diamond.
(TIF)

S4 Fig DAPC of final on-target SNP sub-dataset identifies two clusters. Discriminant analysis of principal components on the final on-target SNP sub-dataset (n = 22). a) the clustering of the samples into two inferred clusters and b) the clustering of samples into the same two inferred clusters as in a), but individuals are identified based on the geographic region from which the sample originated; cluster 1 = Arviat and Victoria Island samples; cluster 2 = Southwestern Alaska samples.
(TIF)

S5 Fig Comparison of identified F_ST_ outlier SNPs using different detection methods. The proportion of F_ST_ outliers identified by each program for a) all identified F_ST_ outlier SNPs from the on-target data and b) the identified F_ST_ outlier SNPs that were retained in the final on-target sub-dataset.
(TIF)

S6 Fig Power analysis results for the final off-target SNP sub-dataset. Assessment of the power for the final off-target SNP sub-dataset (n = 29) with an assumed effective population size of 5000. Chi-squared test results are shown as blue squares and the Fisher exact test results are shown as grey diamonds.
(TIF)

S7 Fig Genome wide F_ST_ estimates between 3 populations of arctic fox in North America. Pairwise Weir and Cockerham F_ST_ values between Southwestern Alaska (Chevak and Hooper Bay), Arviat, and Victoria Island arctic fox populations. Identified outliers are highlighted in red and are those found in S5 Table.
(TIF)

S8 Fig Principal component analysis of the 4 outlier SNPs after linkage-disequilibrium pruning from the off-target dataset. Based on the lack of outliers identified, and the lack of genetic clustering evident by their visualization, we conclude there is no genetic structure among the arctic fox populations based on these off-target data. It is important to note however, that due to the small size of the dataset, and the nature of biallelic markers that conclusions drawn from these data should be done with caution. Arviat (1—Black squares); Chevak (2—Yellow circle); Hooper Bay (3—Light Blue Triangle); Victoria Island (4—Green diamond).
(TIF)

S9 Fig Principal component analysis of the 29 off-target SNPs passing filtering parameters and pruned for linkage disequilibrium. Based on the prominent overlap of all 4 clusters, these data do not suggest genetic structuring. These data were then further investigated with STRUCTURE and DAPC analyses (Fig 2). Arviat (AR–Black circles); Chevak (CV–Yellow circles); Hooper Bay (HB–Light blue circles); Victoria Island (VI–Green circles).
(TIF)

S1 Table Arctic fox sample information. Sample identifiers, year of collection, area of sampling and corresponding arctic rabies variant circulating area of collection.
(XLSX)

S2 Table 116 immunogenetic probe-baited targets enriched for. Describes in reference to the dog genome (per gene): the transcript and gene ID, position in the genome (chromosome, start/stop base pair), number of exons, and the BLASTp hit description.
(XLSX)

S3 Table GATK filtering results for the 96 arctic fox samples. Describes (per sample): the number of raw reads, reads passing GATK filters and those reads not passing the GATK filters due to mapping quality, secondary alignments, and duplicate reads.
(XLSX)

S4 Table Filtered off-target SNP sub-dataset. Describes the position and average coverage for each SNP retained in the final filtered sub-dataset. Filtering parameters were a minor allele frequency threshold = 2% and pruning for linkage disequilibrium.
(XLSX)

S5 Table Identified F_ST_ outliers before and after disequilibrium pruning among arctic fox populations across North America. Describes (per SNP): location, gene association, and predicted gene function in reference to the dog genome. The program identifying the SNP as an F_ST_ outlier is also presented along with an indication of those missense SNP’s with the potential to alter protein function. All BLASTp predicted functions are based upon Canis lupus familiaris unless otherwise specified.
(XLSX)

S6 Table Pairwise F_ST_ 97.5% upper and lower confidence intervals. Outlines the pairwise F_ST_ intervals between the three sampled regions. Interval pairs that were significantly different from zero are bolded.
(XLSX)

S7 Table Comparison of genes under selection based on pN/pS ratios for each sampling region. For each gene highlights the ratio of non-synonymous substitutions per non-synonymous site (pN) to synonymous substitutions per synonymous site (pS) across the three sampled regions, as well as a test of significance using the Chi squared p-values. Bolded ratios indicated those pN/pS ratios greater than or equal to 1 suggestive of directional selection and those pN/pS ratios that have statistically significant p values. Polymorphic sites were determined using SnpEff and potential nonsynonymous/synonymous sites were determined using the coding sequence for each gene as input for DnaSP v6. (Rozas et al. 2017 [68]).
(XLSX)

S1 File Supplementary methods. Additional methods pertaining to linkage disequilibrium pruning and the F_ST_ outlier testing program parameters selected.
(DOCX)
