# Supplementary figures and images for: Genetic structure of immunologically associated candidate genes suggests arctic rabies variants exert differential selection in arctic fox populations
Source: PLoS One. 2021 Oct 29;16(10):e0258975. doi: 10.1371/journal.pone.0258975 (PMC8555846; doi:10.1371/journal.pone.0258975)

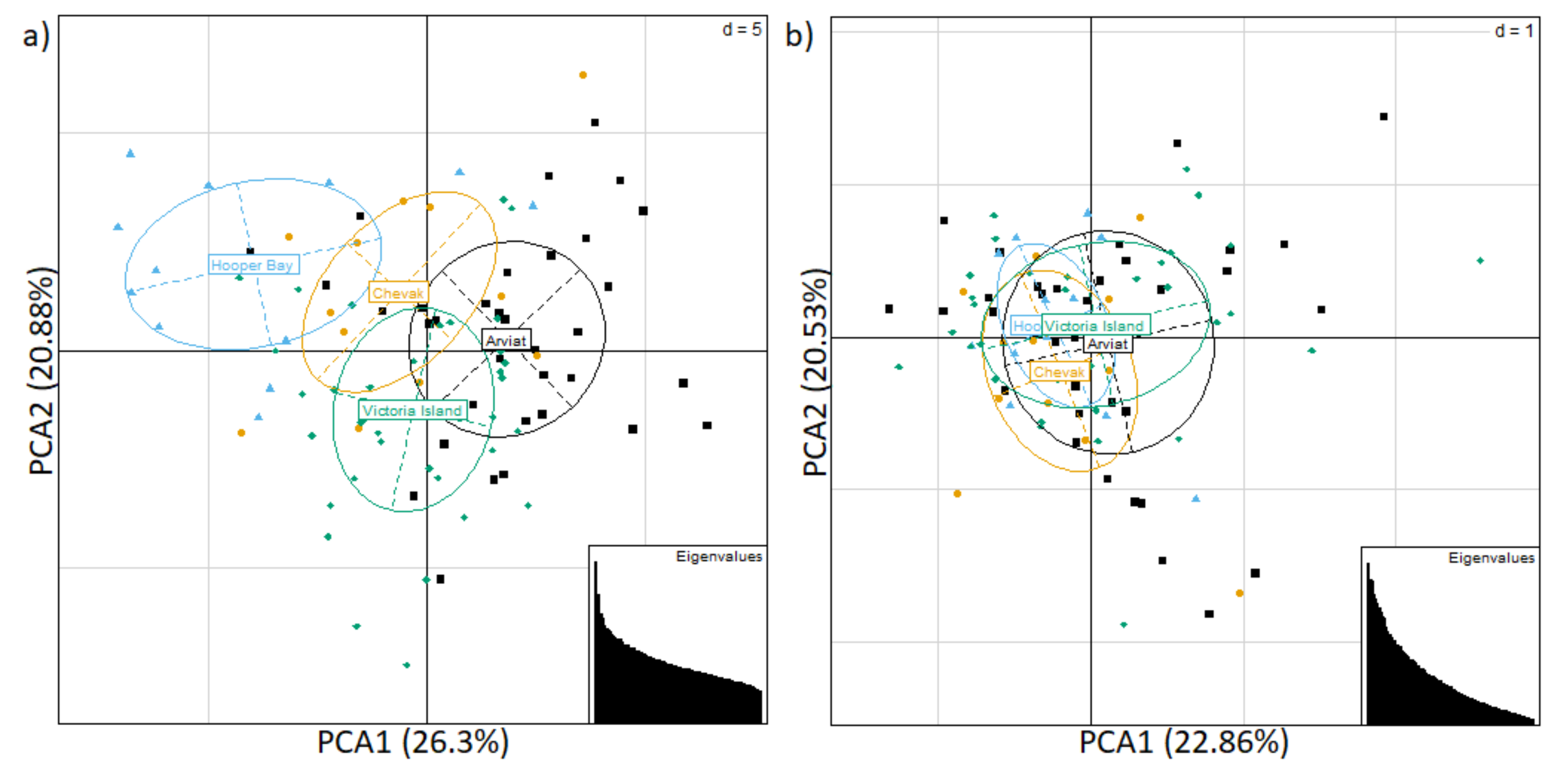

Supplement: S1 Fig — Clustering of the 96 arctic fox samples based on on-target SNPs throughout filtering steps. a) PCA of all off-target SNPs after filtering for MAF, missing-data, and biallelic SNPs (n = 6432) and b) PCA of all identified off-target SNPs after analysis with Variant Effect Predictor and prior to linkage pruning (n = 283). Arviat = black square; Chevak = yellow circle; Hooper Bay = blue triangle; Victoria Island = green diamond. (TIF) [file pone.0258975.s001.tif]

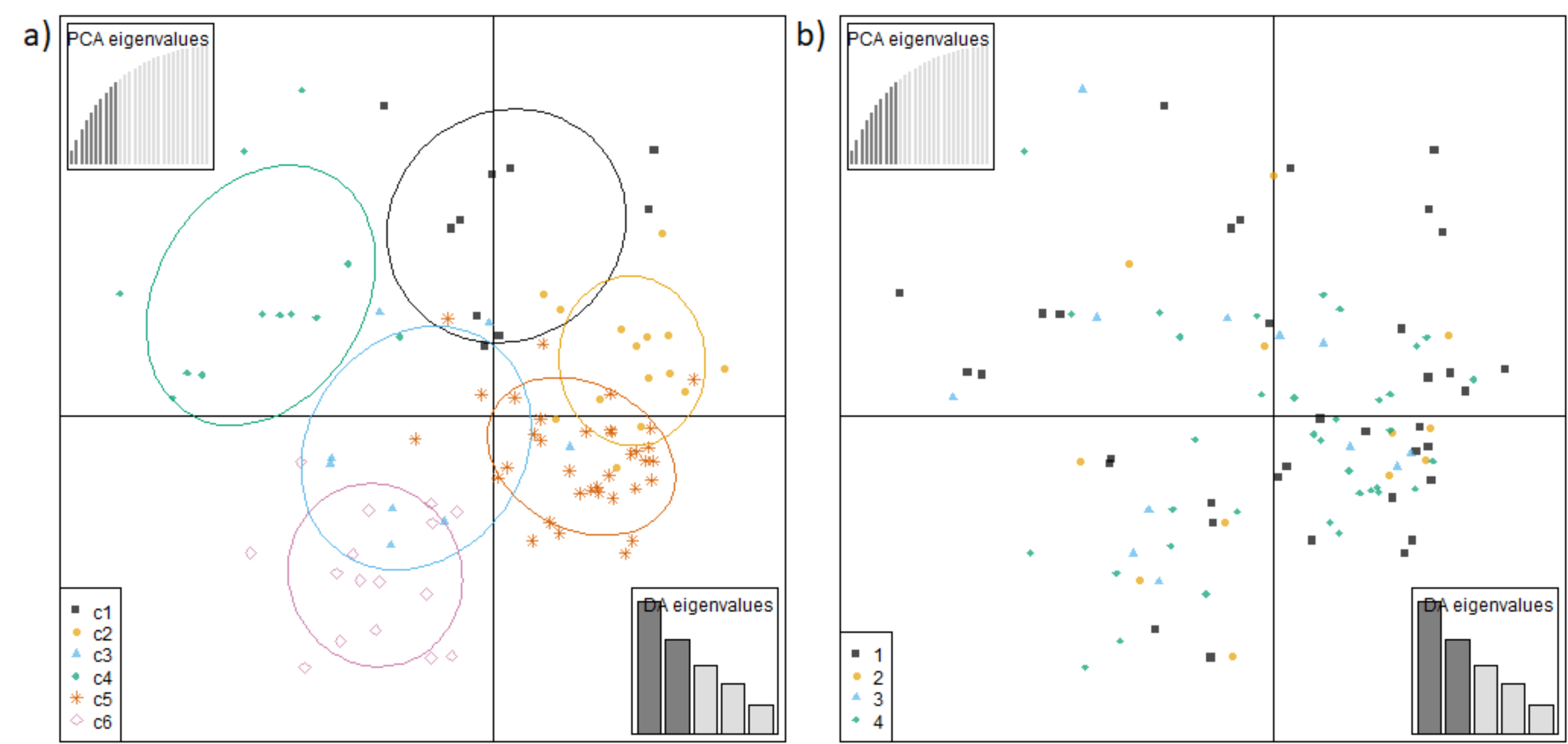

Supplement: S2 Fig — Discriminant analysis of principal components on the final on-target SNP sub-dataset (n = 29). a) the clustering of the samples into six inferred clusters, and b) the clustering of the samples into the same six inferred clusters as in a), but individuals are identified based on the geographical region from which the sample originated; cluster 1 (black square) = Arviat; cluster 2 (yellow circle) = Chevak; cluster 3 (blue triangle)–Hooper Bay; cluster 4 (green diamond)–Victoria Island. (TIF) [file pone.0258975.s002.tif]

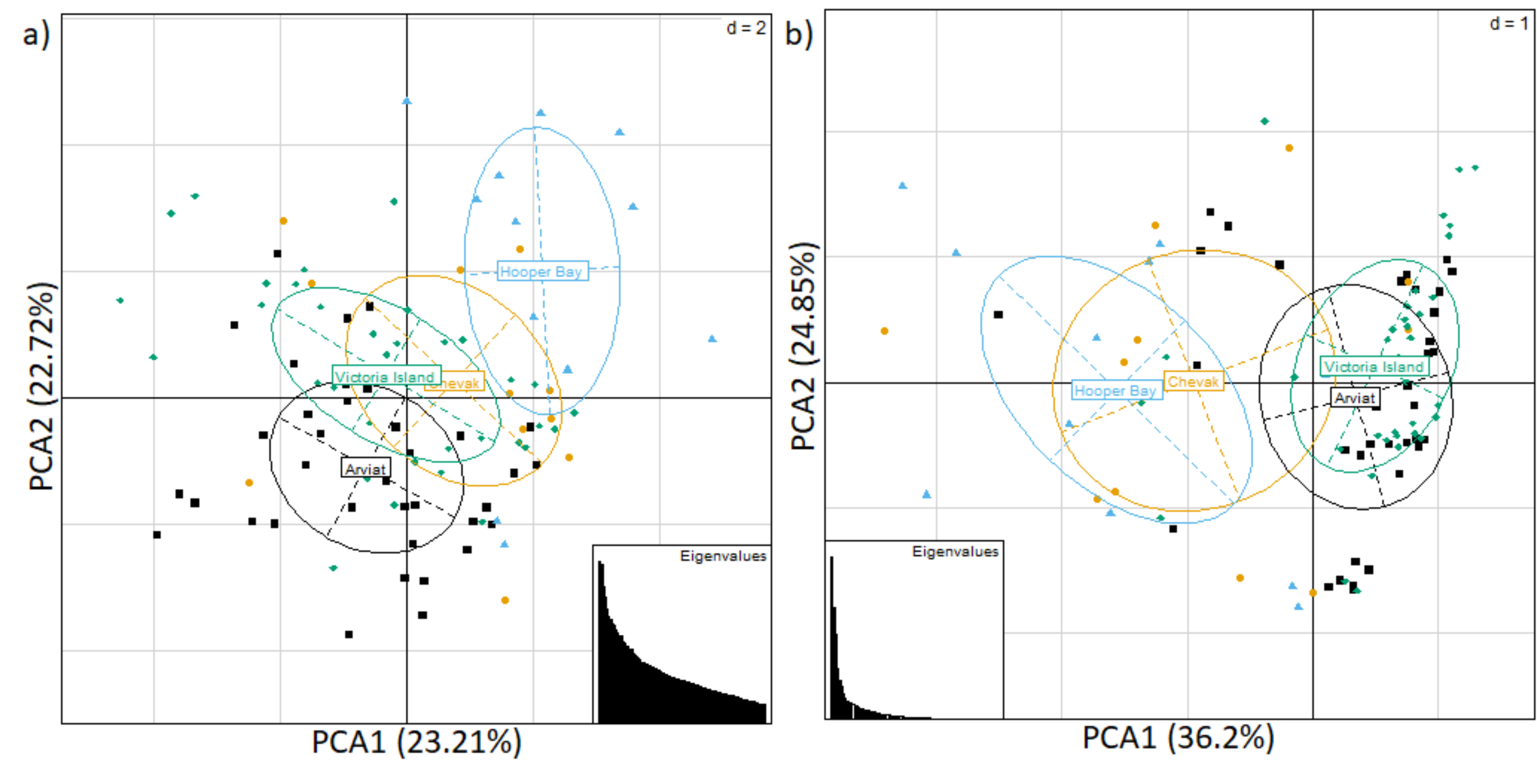

Supplement: S3 Fig — Clustering of the 96 arctic fox samples based on on-target SNPs throughout filtering steps. a) PCA of all on-target SNPs after filtering for MAF, missing-data, and biallelic SNPs (n = 2277) and b) PCA of all identified on-target FST outlier SNPs prior to linkage pruning (n = 107). Arviat = black square; Chevak = yellow circle; Hooper Bay = blue triangle; Victoria Island = green diamond. (TIF) [file pone.0258975.s003.tif]

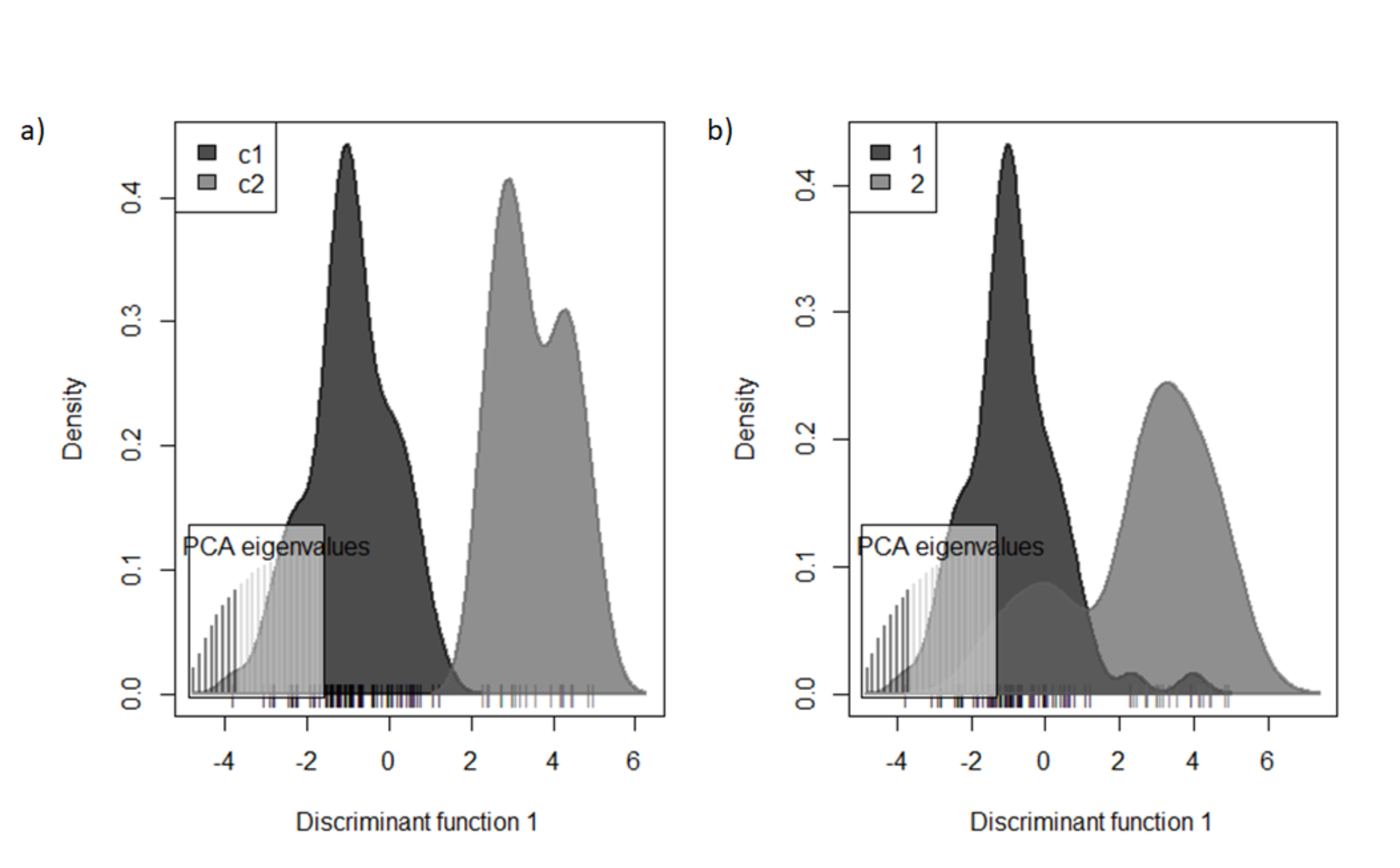

Supplement: S4 Fig — Discriminant analysis of principal components on the final on-target SNP sub-dataset (n = 22). a) the clustering of the samples into two inferred clusters and b) the clustering of samples into the same two inferred clusters as in a), but individuals are identified based on the geographic region from which the sample originated; cluster 1 = Arviat and Victoria Island samples; cluster 2 = Southwestern Alaska samples. (TIF) [file pone.0258975.s004.tif]

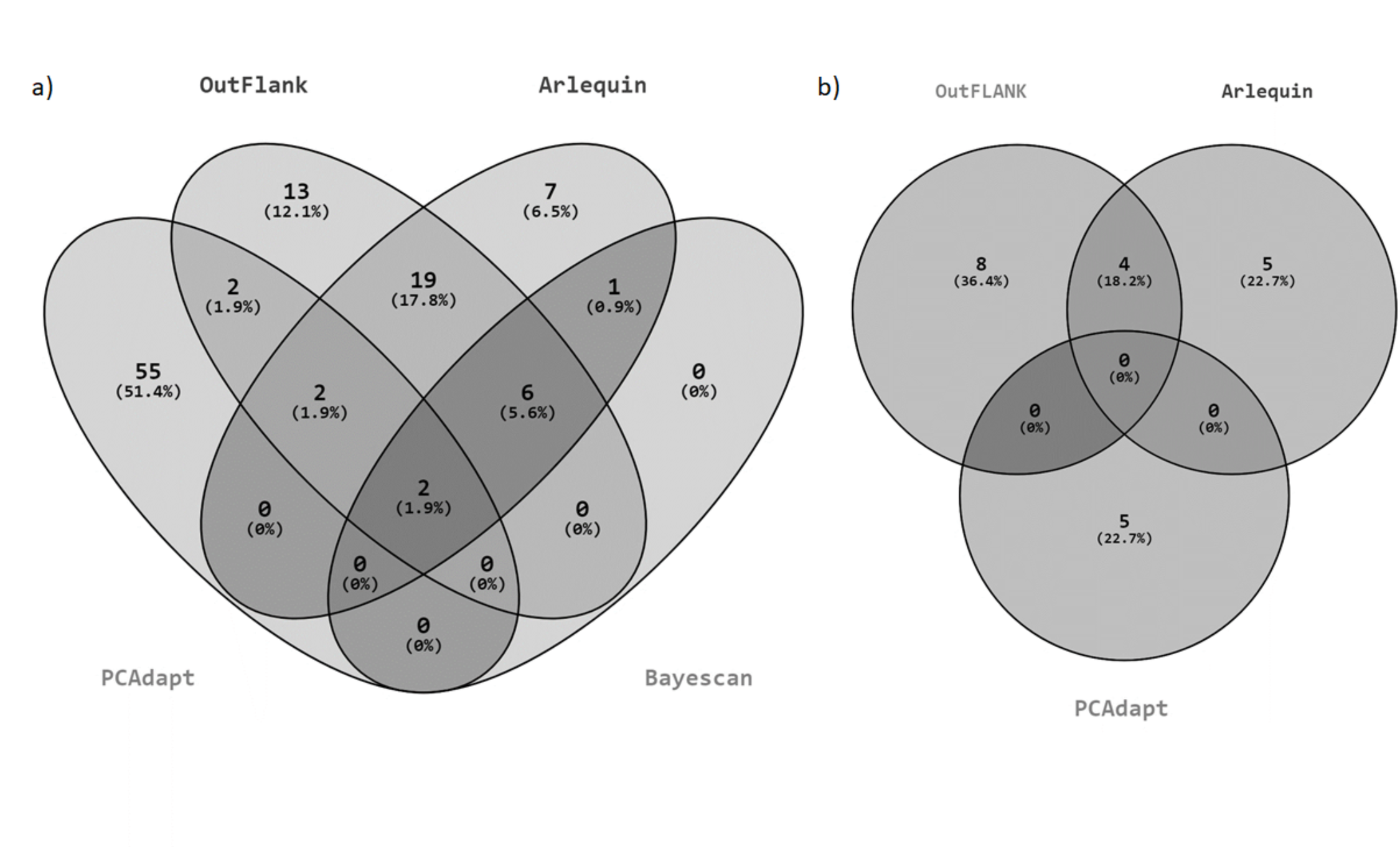

Supplement: S5 Fig — The proportion of FST outliers identified by each program for a) all identified FST outlier SNPs from the on-target data and b) the identified FST outlier SNPs that were retained in the final on-target sub-dataset. (TIF) [file pone.0258975.s005.tif]

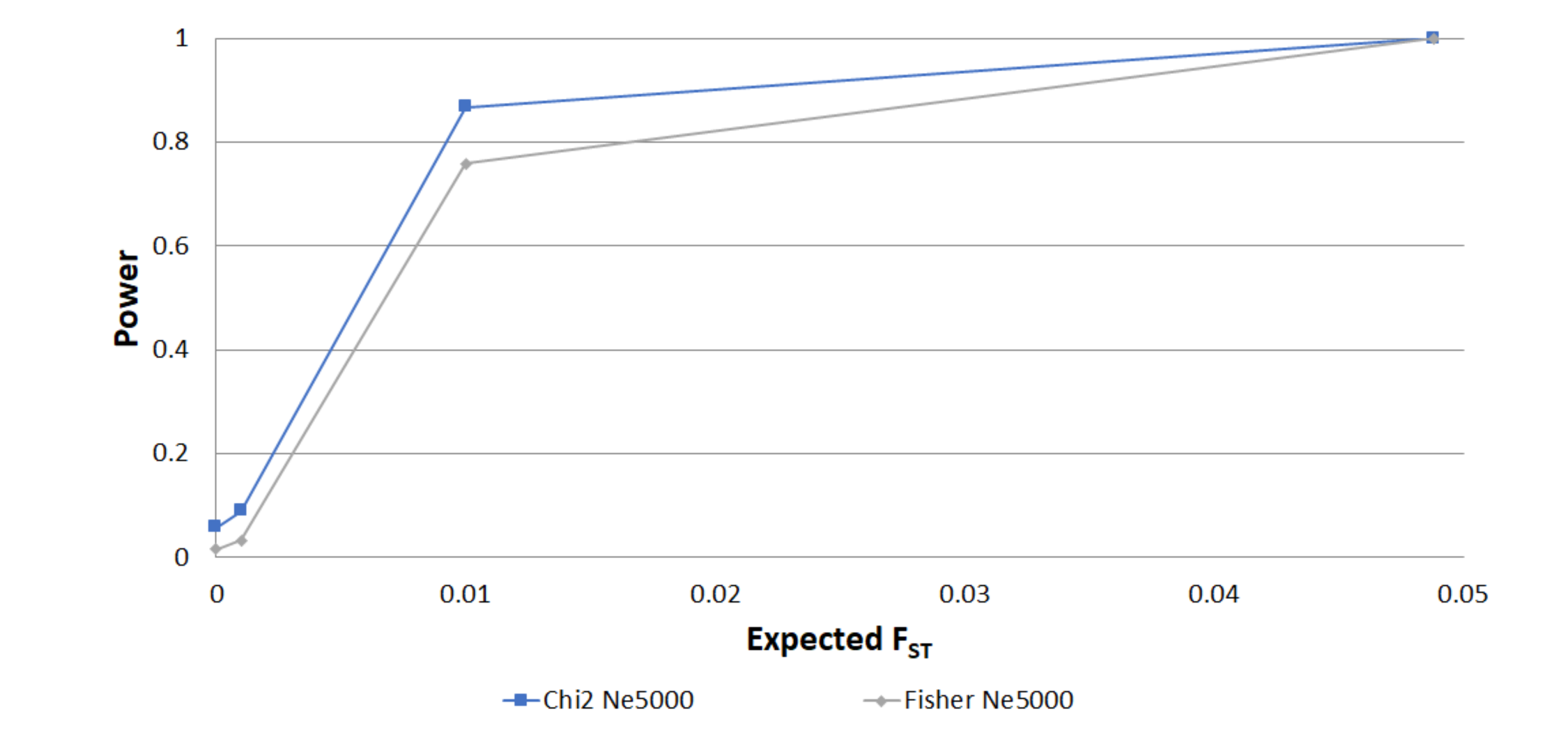

Supplement: S6 Fig — Assessment of the power for the final off-target SNP sub-dataset (n = 29) with an assumed effective population size of 5000. Chi-squared test results are shown as blue squares and the Fisher exact test results are shown as grey diamonds. (TIF) [file pone.0258975.s006.tif]

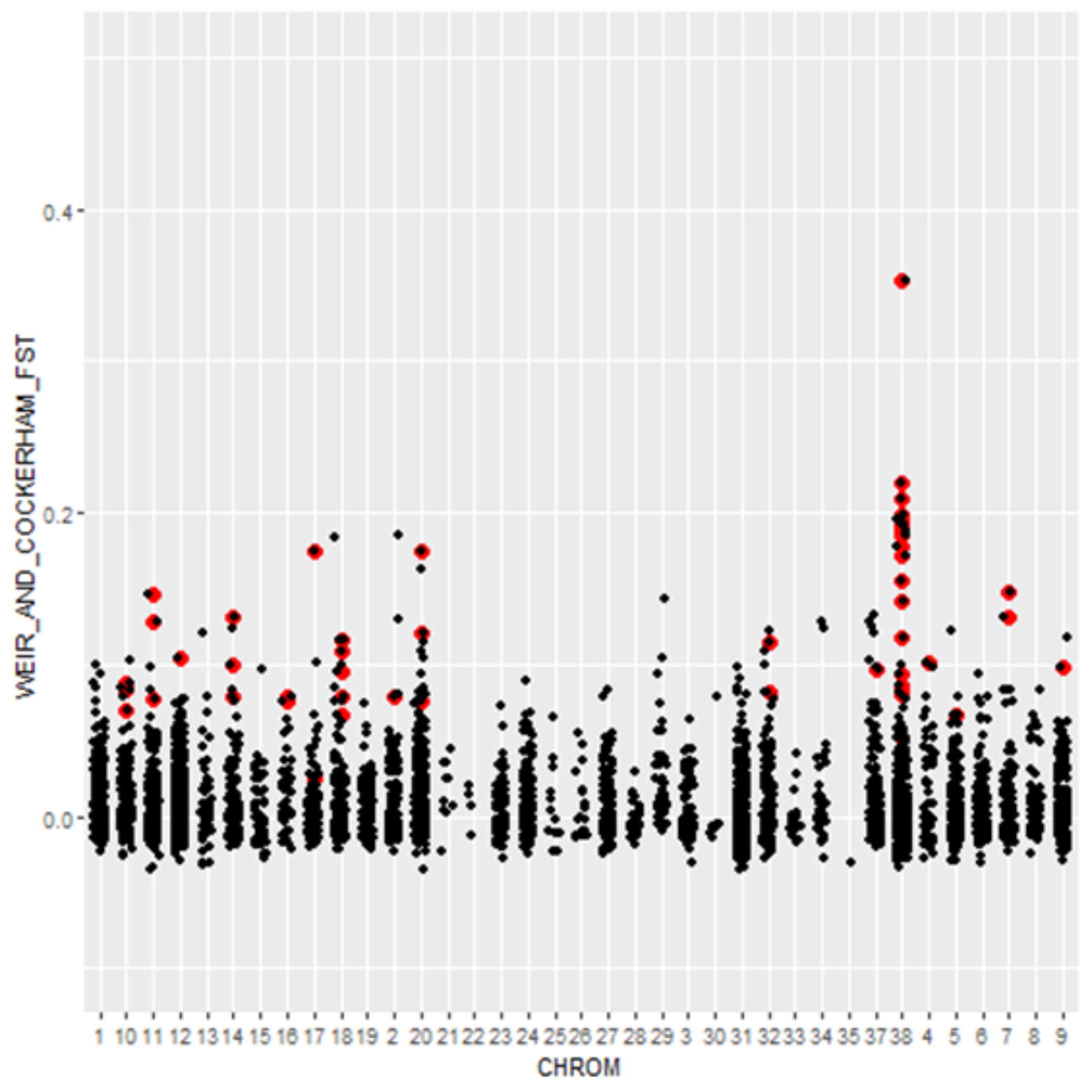

Supplement: S7 Fig — Pairwise Weir and Cockerham FST values between Southwestern Alaska (Chevak and Hooper Bay), Arviat, and Victoria Island arctic fox populations. Identified outliers are highlighted in red and are those found in S5 Table. (TIF) [file pone.0258975.s007.tif]

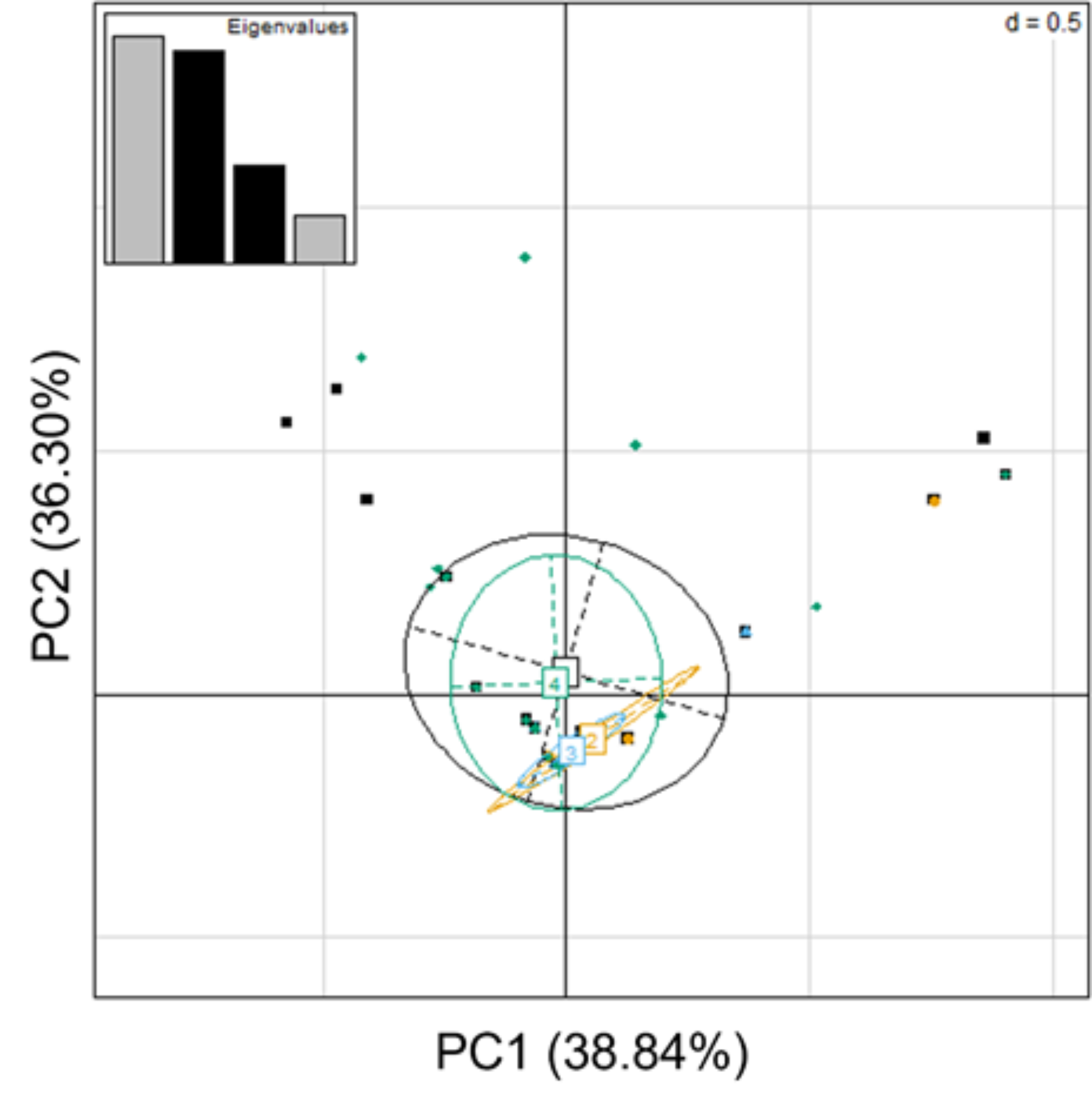

Supplement: S8 Fig — Based on the lack of outliers identified, and the lack of genetic clustering evident by their visualization, we conclude there is no genetic structure among the arctic fox populations based on these off-target data. It is important to note however, that due to the small size of the dataset, and the nature of biallelic markers that conclusions drawn from these data should be done with caution. Arviat (1—Black squares); Chevak (2—Yellow circle); Hooper Bay (3—Light Blue Triangle); Victoria Island (4—Green diamond). (TIF) [file pone.0258975.s008.tif]

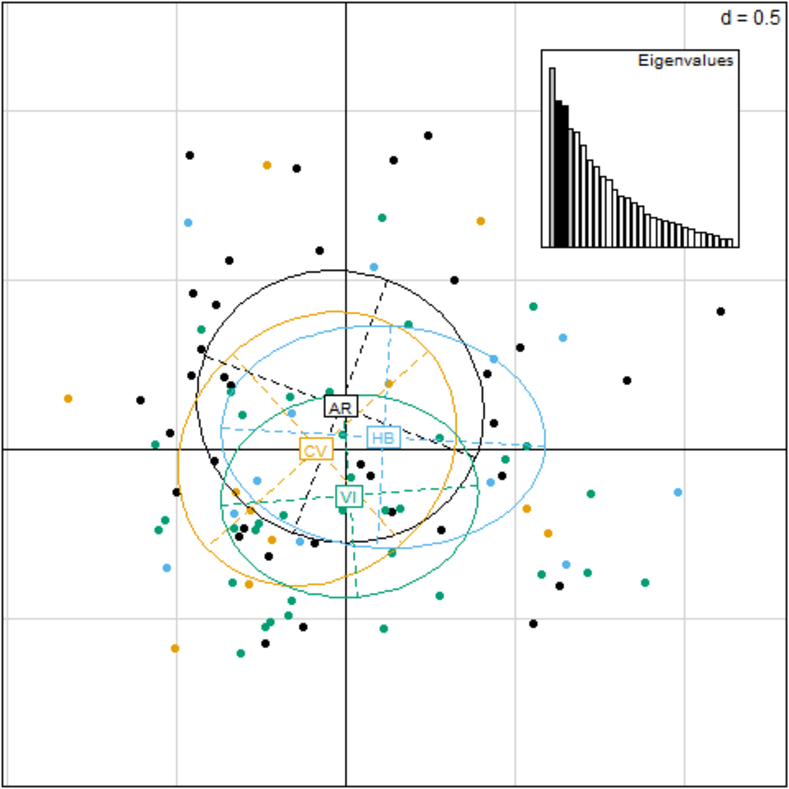

Supplement: S9 Fig — Based on the prominent overlap of all 4 clusters, these data do not suggest genetic structuring. These data were then further investigated with STRUCTURE and DAPC analyses (Fig 2). Arviat (AR–Black circles); Chevak (CV–Yellow circles); Hooper Bay (HB–Light blue circles); Victoria Island (VI–Green circles). (TIF) [file pone.0258975.s009.tif]
