# Supplementary material for: Genetic structure of immunologically associated candidate genes suggests arctic rabies variants exert differential selection in arctic fox populations
Source: PLoS One. 2021 Oct 29;16(10):e0258975. doi: 10.1371/journal.pone.0258975 (PMC8555846; doi:10.1371/journal.pone.0258975)
Supplement: S1 File — Additional methods pertaining to linkage disequilibrium pruning and the FST outlier testing program parameters selected. (DOCX) [file pone.0258975.s017.docx]

# Supplementary Methods

## Linkage Disequilibrium

### SNPRelate Linkage Disequilibrium pruning in R v3.5

library(gdsfmt)

library(SNPRelate)

rm(list = ls())

dev.off()

setwd("PATH:/to/directory")

vcf.fn <- "/PATH/to/filename.vcf"

snpgdsVCF2GDS(vcf.fn, "filename.gds", method="biallelic.only")

(genofile <- snpgdsOpen("filename.gds"))

set.seed(1000)

snpset <- snpgdsLDpruning(genofile, ld.threshold=0.2)

# to get a snp list object --> lists all SNPs analyzed /w locations and Allele Freq.

snplist <- snpgdsSNPList(genofile)

#identify where (Chromsome) the kept snps are

names(snpset)

#get all selected snp id

snpset.id <- unlist(snpset)

#EXPORT

snpgdsGDS2BED(genofile, snp.id=snpset.id, bed.fn="./Export_filename_retained_SNPs")

### Physical Linkage pruning

Filter to ensure LD pruned and retain only a single SNP per 100 kbp window using bcftools v1.9:

bcftools +prune --max-LD 0.2 input_filename.vcf --nsites-per-win 1 --window 100kb --output-type v --output output_filename.vcf

## Outlier Testing Parameters

### PCAdapt v4.0.2

Outlier testing was implemented in RStudio using the pcadapt, vcfR, and qvalue packages. K values of 2-4 were used in conjunction with datasets generated denoting the respective number of clusters. Qvaules were assessed with a threshold of 0.05.

### OutFLANK v0.2

Outlier testing was implemented in RStudio using OutFLANK, ggplot2, and vcfR packages. We used a left- and right- trimfraction of 0.05, minimum expected heterozygosity of 0.1, qthreshold of 0.05, and modified the NumberOfSamples to reflect the number of K-clusters being tested.

### Arlequin v3.5.2.2

Detecting loci under selection was achieved using: 20,000 simulations, 100 demes simulated per group, minimum expected Heterozygosity of zero, maximum expected heterozygosity of one, and assessing pairwise differences. Population comparisons were achieved using: 100 permutations, a significance level of 0.05, a gamma *a* value of zero. Pairwise differences were calculated, and distance matrices were computed. Population differentiation metrics were completed using: genotype frequencies, a 100,000 step Markov Chain, 10,000 dememorization steps, and a significance level of 0.05.

### BayeScan v2.1

We implemented our outlier analysis using Bayescan with: a sample size of 5,000, a thinning interval of 10, 20 pilot runs each with a length of 5,000, a burin in of 50,000, and prior odds for the neutral model of 10.

## References

1. Zheng X, Levine D, Shen J, Gogarten S, Laurie C, Weir B. A High-performance Computing Toolset for Relatedness and Principal Component Analysis of SNP Data. Bioinformatics. 2012; 28(24):3326-3328. doi: 10.1093/bioinformatics/bts606.
2. Danecek P, Bonfield JK, Liddle J, Marshall J, Ohan V, Pollard MO, Whitwham A, Keane T, McCarthy SA, Davies RM, Heng Li H. Twelve years of SAMtools and BCFtools. GigaScience. 2021; 10(2): gib008. doi: 10.1093/gigascience/giab008
3. Luu K, Bazin E, Blum MG. pcadapt: an R package to perform genome scans for selection based on principal component analysis. Molecular ecology resources. 2017 Jan;17(1):67-77.
4. Whitlock MC, Lotterhos KE. Reliable detection of loci responsible for local adaptation: Inference of a null model through trimming the distribution of F ST. The American Naturalist. 2015 Oct 1;186(S1):S24-36.
5. Excoffier L, Lischer HE. Arlequin suite ver 3.5: a new series of programs to perform population genetics analyses under Linux and Windows. Molecular ecology resources. 2010 May;10(3):564-7.
6. Foll M, Gaggiotti O. A genome-scan method to identify selected loci appropriate for both dominant and codominant markers: a Bayesian perspective. Genetics. 2008 Oct 1;180(2):977-93.
